# circRNA-miRNA-mRNA Deregulated Network in Ischemic Heart Failure Patients

**DOI:** 10.3390/cells12212578

**Published:** 2023-11-05

**Authors:** Alisia Madè, Alessia Bibi, Jose Manuel Garcia-Manteiga, Anna Sofia Tascini, Santiago Nicolas Piella, Roman Tikhomirov, Christine Voellenkle, Carlo Gaetano, Przemyslaw Leszek, Serenella Castelvecchio, Lorenzo Menicanti, Fabio Martelli, Simona Greco

**Affiliations:** 1 Molecular Cardiology Laboratory, IRCCS Policlinico San Donato, San Donato Milanese, 20097 Milan, Italy; alisia.made@grupposandonato.it (A.M.); alessia.bibi@grupposandonato.it (A.B.); santiagonicolas.piella@grupposandonato.it (S.N.P.); roman.tikhomirov@manchester.ac.uk (R.T.); christine.voellenkle@grupposandonato.it (C.V.); simona.greco@grupposandonato.it (S.G.); 2 Department of Biosciences, University of Milan, 20122 Milan, Italy; 3 Center for Omics Sciences COSR, BioInformatics Laboratory, San Raffaele Scientific Institute, 20132 Milan, Italy; garciamanteiga.josemanuel@hsr.it (J.M.G.-M.); tascini.anna@hsr.it (A.S.T.); 4 Università Vita-Salute San Raffaele, 20132 Milan, Italy; 5 Laboratory of Epigenetics, Istituti Clinici Scientifici Maugeri IRCCS, 27100 Pavia, Italy; carlo.gaetano@icsmaugeri.it; 6 Department of Heart Failure and Transplantology, National Institute of Cardiology, 04-628 Warsaw, Poland; przemyslaw.leszek@ikard.pl; 7 Department of Adult Cardiac Surgery, IRCCS Policlinico San Donato, San Donato Milanese, 20097 Milan, Italy; serenella.castelvecchio@grupposandonato.it (S.C.); lorenzo.menicanti@grupposandonato.it (L.M.)

**Keywords:** circular RNA, noncoding RNA, heart failure, ceRNA network, circRNA, circBPTF

## Abstract

Noncoding RNAs (ncRNAs), which include circular RNAs (circRNAs) and microRNAs (miRNAs), regulate the development of cardiovascular diseases (CVD). Notably, circRNAs can interact with miRNAs, influencing their specific mRNA targets’ levels and shaping a competing endogenous RNAs (ceRNA) network. However, these interactions and their respective functions remain largely unexplored in ischemic heart failure (IHF). This study is aimed at identifying circRNA-centered ceRNA networks in non-end-stage IHF. Approximately 662 circRNA-miRNA-mRNA interactions were identified in the heart by combining state-of-the-art bioinformatics tools with experimental data. Importantly, KEGG terms of the enriched mRNA indicated CVD-related signaling pathways. A specific network centered on circBPTF was validated experimentally. The levels of let-7a-5p, miR-18a-3p, miR-146b-5p, and miR-196b-5p were enriched in circBPTF pull-down experiments, and circBPTF silencing inhibited the expression of *HDAC9* and *LRRC17*, which are targets of miR-196b-5p. Furthermore, as suggested by the enriched pathway terms of the circBPTF ceRNA network, circBPTF inhibition elicited endothelial cell cycle arrest. circBPTF expression increased in endothelial cells exposed to hypoxia, and its upregulation was confirmed in cardiac samples of 36 end-stage IHF patients compared to healthy controls. In conclusion, circRNAs act as miRNA sponges, regulating the functions of multiple mRNA targets, thus providing a novel vision of HF pathogenesis and laying the theoretical foundation for further experimental studies.

## 1. Introduction

Non-coding RNAs (ncRNAs) [1,2] are transcripts that are not translated into proteins. They have been recognized to have critical regulatory functions [3,4,5], and microRNAs (miRNAs), long noncoding RNAs (lncRNAs), and circular RNAs (circRNAs) are the most studied ncRNA classes. In particular, miRNAs are short ncRNAs (18–25 nucleotides) that can modulate gene expression by binding to MicroRNA Responsive Elements (MREs) present on target transcripts [6]. Increasing data have shown the association of miRNAs with human cardiovascular diseases (CVDs) [7,8,9]. In particular, a large number of transcriptomic studies have identified specific miRNAs differentially expressed (DE) in the left ventricles (LV) of both dilated and ischemic cardiomyopathy patients [10,11,12]. Indeed, our previous data, obtained from the LV miRNA profiling of non-end-stage ischemic heart failure (IHF) patients and healthy subjects [13], identified 17 miRNAs that were modulated in diabetic IHF or non-diabetic IHF patients when compared with control subjects and 6 DE miRNAs in diabetic versus non-diabetic IHF patients.

circRNAs were described for the first time in 1976 by Sanger et al. as plant viroids containing covalently closed circular RNA molecules [14]. Nowadays, in eukaryotic cells, the term circRNA identifies a single-strand covalently closed molecule generated through the back-splicing of linear mRNAs [15]. In the back-splicing mechanism, a 3’ exon of a gene is back-spliced to a 5’ exon, resulting in a circular product without 5’ to 3’ polarity, free ends, 5’ caps, and 3’ poly-A structures [16,17]. Evidence from ever-increasing high-throughput transcriptome sequencing programs has identified circRNAs as abundant and stable transcripts in different cell lines and tissues [18]. Accordingly, circRNAs are significantly more stable than their linear counterparts [19]. Indeed, circRNAs are resistant to exonucleases, including RNAse R, due to the lack of a free end [20]. circRNA functions are related to their cellular location. For example, exonic circRNAs (i.e., not containing intronic sequences) are mostly cytoplasmic and, in this compartment, can interact with miRNAs. Therefore, circRNAs can operate as competing endogenous RNAs (ceRNAs), acting as miRNA sinks or sponges, thus actively competing with protein-coding genes for the same pool of miRNAs [21,22].

Indeed, miRNAs bind to MREs on the target RNA transcripts by partial complementarity, which results in the repression of target gene expression [23,24]. Given the presence of multiple MREs on each mRNA, a single miRNA can repress hundreds of transcripts that regulate multiple biological and pathological processes [6,25,26]. According to this model, miRNAs can be considered active regulatory ncRNAs, while mRNAs play a more passive role. However, this perspective is undergoing a reevaluation due to recent findings. It has been demonstrated that lncRNAs, including circRNAs and pseudogenes, can act as competitors of miRNAs, thus actively competing with protein-coding genes for the same pool of miRNAs [22,27,28]. These ceRNAs essentially act as "sponges," disrupting the normal targeting function of mRNAs [29,30,31].

Another important function of cytoplasmic circRNAs is their ability to interact with RNA-binding proteins, a group of heterogeneous proteins involved in RNA processing events such as maturation, transport, and translation [32].

Following the progress in high-throughput technologies, thousands of circRNAs have been identified in human, rat, and mouse cardiac tissues [32,33,34]. A large amount of these circRNAs showed host gene-independent expression derived from cardiac genes involved in heart development, membrane trafficking, and muscle structure development [35]. Moreover, many circRNAs have been reported to be differentially expressed in human-diseased hearts compared to healthy controls [33,34,36,37] and in mice with myocardial infarction-induced HF or diabetes-induced cardiomyopathies [38,39]. However, there are incomplete and sometimes contradictory results on circRNA regulation and function in HF, possibly due to the heterogeneity and small number of patients analyzed, indicating that the understanding of the role of circRNAs in HF physio-pathology is still far from complete.

On account of these data, a deep knowledge of the functional interactions among the different families of coding and non-coding RNAs may help shed light on the phenotypic consequences of the transcriptomic dysregulations in the HF process. Indeed, the very few data on circRNA-miRNA-mRNA networks in IHF [40,41,42] are limited to end-stage patients.

In this study, the combination of bioinformatics predictions with experimental data obtained in IHF patients allowed the identification of circRNA-centered ceRNA networks in failing hearts. A specific network centered on circBPTF was validated experimentally, confirming both the identified molecular interactions and their functional consequences. Thus, the present study provides an example of a circRNA-miRNA-mRNA network based on the ceRNA theory in non-end-stage IHF patients.

## 2. Materials and Methods

### 2.1. Patient Selection and Tissue Collection

For non-end-stage IHF studies, LV cardiac biopsies were harvested at IRCCS Policlinico San Donato from patients affected by dilated hypokinetic ischemic cardiomyopathy during the Surgical Ventricular Reconstruction procedure [43]. Biopsies were collected from the non-ischemic and dysfunctional remote myocardium, immediately immersed in RNAlater (Qiagen, Venlo, The Netherlands), and stored at 4 °C for <24 h before RNA extraction.

LV end-stage (ES) IHF biopsies were collected at the Department of Heart Failure and Transplantology, Cardinal Stefan Wyszyński Institute of Cardiology, Warszawa, Poland, from ES IHF patients undergoing heart transplantation. Healthy human LV was obtained from age- and sex-matched organ donor patients whose hearts were not used for transplantation due to technical reasons (e.g., donor/recipient incompatibility) and collected at the Department of Heart Failure and Transplantology, Department of Mechanical Circulatory Support and Transplant, National Institute of Cardiology, Warszawa, Poland. The donors had no relevant cardiological history or abnormalities in ECG or echocardiography. Samples were rinsed immediately in saline, blotted dry, frozen in liquid nitrogen, and kept at −80 °C until further processing.

The protocols were authorized by local Ethics Committees (ASL MilanoDue Ethics Committee, protocol number: 2438; 27 January 2009; San Raffaele Hospital Ethics Committee, protocol number: 85/int/2016; 9/6/2016; and Terenowej Komisji Bio-etycznej Przy Instytucie Kardiologii-Warsaw Ethics Committee, protocol number: IK-NPIA-0021-14/1426/18). IHF patients and control characteristics are described in Supplementary Table S1.

### 2.2. RNA-Sequencing

Twenty samples from non-end-stage IHF patients and 19 healthy controls were used to profile the transcriptome using RNA-sequencing (RNA-Seq). Total RNA libraries from all the samples were prepared in three batches using the ribominus stranded protocol (TruSeq Stranded Total RNA- rRNA, Illumina, San Diego, CA, USA) and sequenced using the NovaSeq/NextSeq Illumina (San Diego, CA, USA) sequencing platforms in pair-end mode (2 × 100/2 × 150 bp) with an average coverage of 90M fragments. Reads were processed with an in-house pipeline. Low-quality bases and adapters were trimmed with Trimmomatic (v0.39), aligned to the hg38 human assembly using STAR (v2.5.3a) [44], and counts mapped to genes produced using featureCounts (v1.6.4) with the basic annotation from Gencode (v31). Differential gene expression was made using DESeq2 (v1.22.1) [45] with a statistical analysis that included the three batches as covariates in the statistical design. An adjusted *p*-value < 0.01 was calculated with the Benjamini-Hochberg FDR method [46] and used as a threshold. The calculated power was 0.85, considering a nominal alpha of 0.05, an FDR correction of 0.01, and the actual percentage of differentially expressed genes, dispersion per gene, and log_2_ fold change as calculated by DESeq2, considering a delta for biologically interesting genes of log_2_ fold change greater than one [47]. Differential expression was visualized by a heatmap obtained using ClustVis (https://biit.cs.ut.ee/clustvis/ accessed on 14 June 2022) with hierarchical clustering expressed by Pearson correlation.

### 2.3. circRNA Quantification and Differential Expression Analysis

circRNAs were quantified with CIRIquant v1.2 [48] using the same genome build and annotation as for the RNA-seq quantification of the linear transcripts. According to the CIRIquant recommended pipeline, circRNA identification was performed with CIRI2; then, to increase accuracy, generated pseudo-reference sequences for the identified circRNA transcripts were re-aligned to putative BSJ (back-spliced-junction) reads. The BSJ expression level matrix was produced from the generated GTF files with the “prep_CIRIquant” of the CIRIquant toolkit. A total of 134,712 unique back-splice sites were identified across all libraries. A data abundance check filter was applied, and only circRNAs displaying ≥ 5 reads in at least 50% of the samples were considered for further analysis, obtaining 1500 back-splice junctions.

Differential BSJ expression was performed with limma voom [49], using the total number of BSJ reads for library size and gene-level normalization factors. The preparation batches were included in the differential expression design to remove potential batch effects. A canonical *p*-value < 0.05 was used as a statistical significance threshold, obtaining 213 back-splice junctions differentially expressed in IHF vs. controls.

### 2.4. Construction of the circRNA-miRNA-mRNA Network

In order to identify the circRNA-miRNA interactions, we interrogated the MREs present in the sequences of the 16 validated circRNAs by using the Encyclopedia of RNA Interactomes (ENCORI) [50] (https://starbase.sysu.edu.cn/index.php, accessed on 7 April 2023), the Circular RNA Interactome (CircInteractome) [51] (https://circinteractome.nia.nih.gov/mirna_target_sites.html, accessed on 7 April 2023), and the CircAtlas 2.0 [52] databases that integrate large-scale Argonaute CLIP-Seq data (ENCORI) and miRNA targets prediction algorithms (CircInteractome and CircAtlas). MirDIP 4.1 [53] (https://ophid.utoronto.ca/mirDIP/, accessed on 7 April 2023) integrating predictions across multiple resources was used for target identification with “high confidence” and “prediction by ≥5 resources” filters. The circRNA-miRNA-mRNA network was built up using Cytoscape version 3.5.1 (http://www.cytoscape.org, accessed on 7 April 2023). The Gene Ontology analysis for biological processes and molecular functions was performed using Enrichr (https://maayanlab.cloud/Enrichr/, accessed on 7 April 2023) [54] with default parameters. Kyoto Encyclopedia of Genes and Genomes (KEGG) pathway enrichment analysis was visualized using ShinyGO v0.75 [55] (http://bioinformatics.sdstate.edu/go/, accessed on 7 April 2023) and Enrichr (https://maayanlab.cloud/Enrichr/, accessed on 7 April 2023) [54] with default parameters.

### 2.5. microRNA Pull-Down Experiments

miR-196b-5p AntiSense Oligonucleotides (ASO) and negative control ASO (NEG CTR-ASO), with 3′-biotin labeling, were synthesized by Eurofins Genomics (Ebersberg, Germany) and used for miRNA pull-down assays. ASO sequences are reported in Supplementary Table S2B. Sub-confluent HUVEC cells were transfected as indicated in the Supplementary Methods, and after 48 h cells were lysed with 700 μL of ice-cold polysome extraction buffer (20 mM Tris-HCl, pH 7.5, 100 mM KCl, 5 mM MgCl2, and 0.5% Nonidet P-40) supplemented with 40 U of RNase inhibitor (Promega, Madison, WI, USA) and 5 μL of 20× protease inhibitor (Thermo Fisher Scientific Inc, Waltham, MA, USA). Next, samples were incubated on ice for 20 min and then centrifuged at 10,000× *g* for 15 min at 4 °C. The cell lysates were incubated with 50 μL of streptavidin beads (Thermo Fisher Scientific Inc.), 40 U of RNase inhibitor (Promega, Madison, WI, USA)), and 5 μL of 20× protease inhibitor (Thermo Fisher Scientific Inc., Waltham, MA, USA) on a tube rotator overnight at 4 °C. The next day, after 5 washing steps, TRIzol reagent (Thermo Fisher Scientific Inc., Waltham, MA, USA) was added for RNA extraction.

### 2.6. Circular RNA Pull-Down Experiments

circBPTF-ASOs targeting the circBPTF back-splice junction sequence and negative control oligonucleotides (NEG CTR-ASO) [56], with 3′-biotin labeling, were synthesized by Eurofins Genomics (Ebersberg, Germany) and used for the circRNA pull-down assay as described by Das et al. [56]. ASO sequences are reported in Supplementary Table S2B. Briefly, 2.5 × 10^6^ HUVEC were lysed with 1 mL of ice-cold polysome extraction buffer (20 mM Tris-HCl, pH 7.5, 100 mM KCl, 5 mM MgCl2, and 0.5% Nonidet P-40) and centrifuged at 12,000× *g* for 10 min at 4 °C. The supernatant was incubated on a tube rotator with 1 μL of 100 μM circBPTF-ASO or NEG CTR-ASO overnight at 4 °C. The next day, 50 μL of streptavidin beads (Thermo Fisher Scientific Inc., Waltham, MA, USA), 40 U of RNase inhibitor (Promega, Madison, WI, USA), and 5 μL of 20× protease inhibitor (Thermo Fisher Scientific Inc., Waltham, MA, USA)) were added to the mixture and incubated for 90 min on a tube rotator. After five washing steps, TRIzol reagent (Thermo Fisher Scientific Inc., Waltham, MA, USA) was added for RNA extraction.

### 2.7. Statistical Analysis

Continuous variables were expressed as the mean ± standard error of the mean (SEM). The Mann–Whitney or unpaired *t*-test was used for group-wise comparisons. ANOVA, or *t*-test assay corrected for multiple comparisons, were used as appropriate. All tests were performed two-sided, and a *p* < 0.05 was considered statistically significant. GraphPad Prism v.8.3.0 software (GraphPad Software Inc., Boston, MA, USA) was used for statistical analysis.

## 3. Results

### 3.1. Workflow of the circRNA-miRNA-mRNA Network Construction

**Figure 1:**
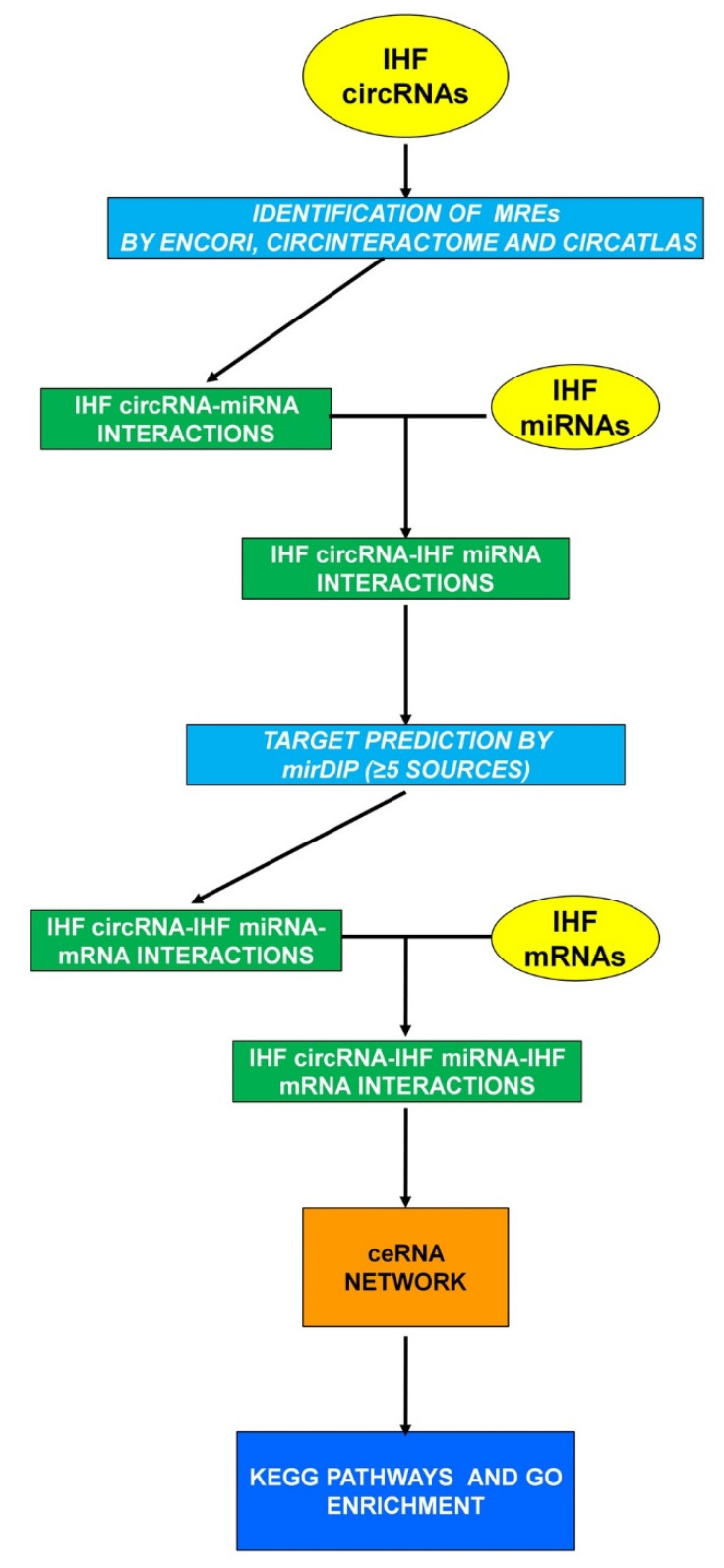
circRNA-miRNA-mRNA network workflow. Experimental datasets are indicated in yellow ovals, bioinformatics analyses in light-blue squares, and RNA interactions in green squares to yield a ceRNA network (orange square) characterized by functional enrichment analysis of KEGG and GO terms (dark blue square).

In order to identify a large-scale regulatory network among multiple RNA types constituted by circRNAs, miRNAs, and mRNAs in non-end-stage IHF patients, both bioinformatics and experimental analyses were performed following the workflow shown in Figure 1. A particularly stringent pipeline was adopted, taking advantage of independent techniques. This may introduce some biases and restrict the number of interactions identified, but the aim was to maximize specificity over sensitivity. The transcriptomic dysregulations characterizing the myocardium of non-end-stage IHF patients were evaluated experimentally. DE mRNAs (IHF DE mRNAs) and circRNAs (IHF DE circRNAs) were assessed by analyzing a newly generated transcriptomic dataset; a dataset including patients with similar characteristics was used to identify DE miRNAs (IHF DE miRNAs) [13]. The bioinformatics analysis identified miRNAs potentially interacting with the dysregulated circRNAs; these miRNAs were filtered for their dysregulation in IHF, assuming that the circRNA-miRNA interaction would lead to a counter-regulation [30,31,57]. Then, a bioinformatics analysis was used again to identify mRNAs potentially interacting with the selected miRNAs, and a second experimental filter was imposed, considering only those mRNAs displaying an opposite regulation with their targeting miRNAs. The resulting mRNAs were considered components of the circRNAs-miRNA-mRNA ceRNA-network characterizing IHF and were further analyzed for pathway enrichment analysis.

### 3.2. Differentially Expressed miRNAs in the Left Ventricle of Non-End-Stage IHF Patients

Previous results were obtained by analyzing LV samples of 29 non-end-stage IHF patients and 16 healthy individuals [13] (Supplementary Table S1A) and identifying DE miRNAs in diabetic vs. non-diabetic IHF patients and controls. In order to obtain a list of DE miRNAs in IHF, the dataset comparing IHF patients with controls, regardless of their diabetes status, was re-analyzed. After a *t*-test with multiple comparison correction, the levels of 18 and 31 miRNAs proved to be increased or decreased, respectively, in IHF (Supplementary Table S3B). These data provided an IHF miRNA signature for further use in the network design.

### 3.3. Differentially Expressed mRNAs in the Left Ventricle of Non-End-Stage IHF Patients

**Figure 2:**
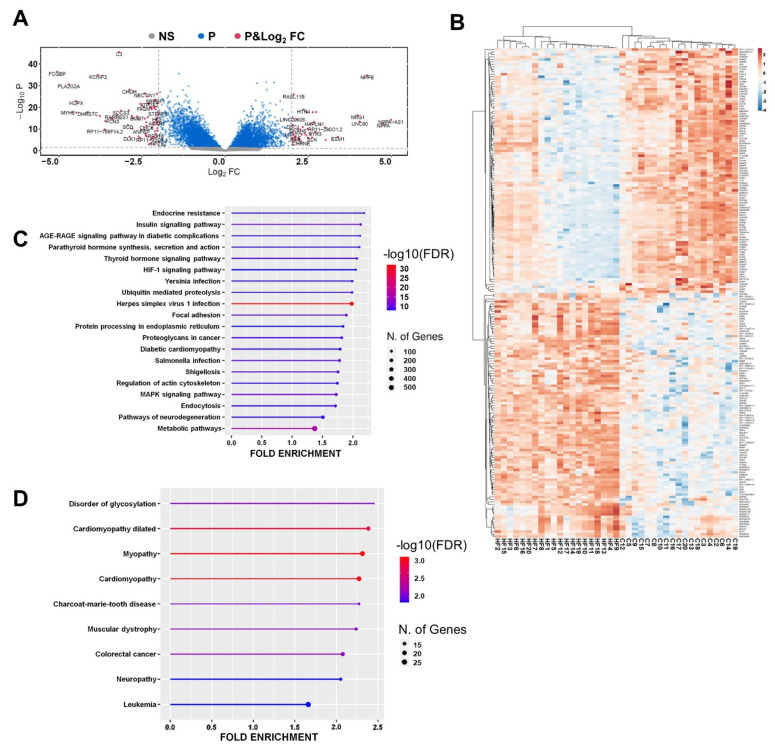
mRNA is differentially expressed in non-end-stage IHF patients. (**A**) Volcano plot of DE mRNAs between IHF and controls Vertical and horizontal dotted lines delimit red plots (*p* and log_2_ fold change) representing DE mRNAs with *p*adj < 0.01 and log_2_ Fold Change >|1.0|, blue (P) plots representing mRNAs displaying a significant DE with *p*adj ≥ 0.01 but with a log_2_ fold change < |1.0, and grey (NS) displaying no significant DE. (**B**) Heatmap showing each patient and control values expressed as Pearson’s correlation coefficient. The unsupervised clustering showed a perfect segregation of IHF from control samples. (**C**,**D**) Enriched top 20 KEGG pathways (**C**) and enriched OMIM diseases (**D**) from IHF DE mRNAs are represented by lollipop graphs. The size of the lollipop indicates the number of genes, and colors indicate the statistical significance (red for lower FDR, blue for higher FDR). For details on DE mRNAs, please refer to Supplementary Table S4A.

The second step for network building was the mRNA profiling by RNA-Seq of LV RNA samples from 20 non-end-stage IHF patients and 20 healthy controls (Supplementary Table S1B). Samples were harvested from the myocardial area, remote from the scar, to assess the molecular processes underpinning HF remodeling. After filtering and normalizing raw data using *p*adj < 0.01 as a threshold, a total of 7,172 DE mRNAs (3,366 up- and 3,806 down-regulated) were identified in IHF patients (Supplementary Table S4A). Figure 2A shows a volcano plot of the DE mRNAs displaying, as expected, *NPPA, NPPB, NPPA-AS1, NRG1,* and *KCNIP2, HOPX* and *MYH6* among the most upregulated and downregulated mRNAs, respectively, validating the analysis [58,59,60,61,62,63,64].

An unsupervised cluster analysis was performed on the 200 top significant DE transcripts between IHF and controls (*p* < 0.01 and >|1| as log_2_ fold change), and Pearson correlation indicated a perfect discrimination between IHF and control samples (Figure 2B).

Interestingly, functional enrichment analysis of KEGG terms indicated among the overrepresented pathways, Insulin, Thyroid, Diabetic complications, and Diabetic Cardiomyopathy pathways, as well as HIF-1, Ubiquitin proteolysis, and Mitogen-Activated Protein Kinase (MAPK) signaling (Figure 2C). In addition, the Online Mendelian Inheritance in Man (OMIM) ontology analysis identified Dilated Cardiomyopathy, Myopathy and Cardiomyopathy among the enriched disease terms (Figure 2D). Gene Ontology analysis indicated, as enriched Biological Processes terms, regulation of DNA-templated Transcription, Protein Ubiquitination, Response to Transforming Growth Factor Beta (Supplementary Table S4B), and as enriched Molecular Functions terms, Ubiquitin-Protein Transferase Activity, Methylated Histone Binding, Histone Deacetylase Binding (Supplementary Table S4C).

Thus, mRNAs that were DE in IHF were identified, indicating the enrichment of several KEGG and GO terms relevant for cardiac disease pathogenesis.

### 3.4. Differentially Expressed circRNAs in the Left Ventricle of Non-End-Stage IHF Patients

**Figure 3:**
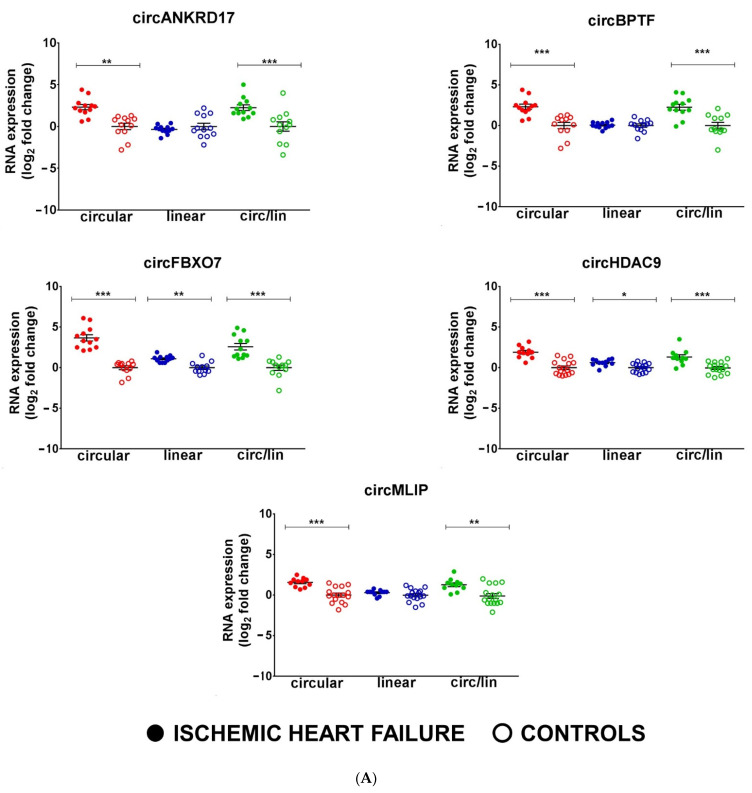

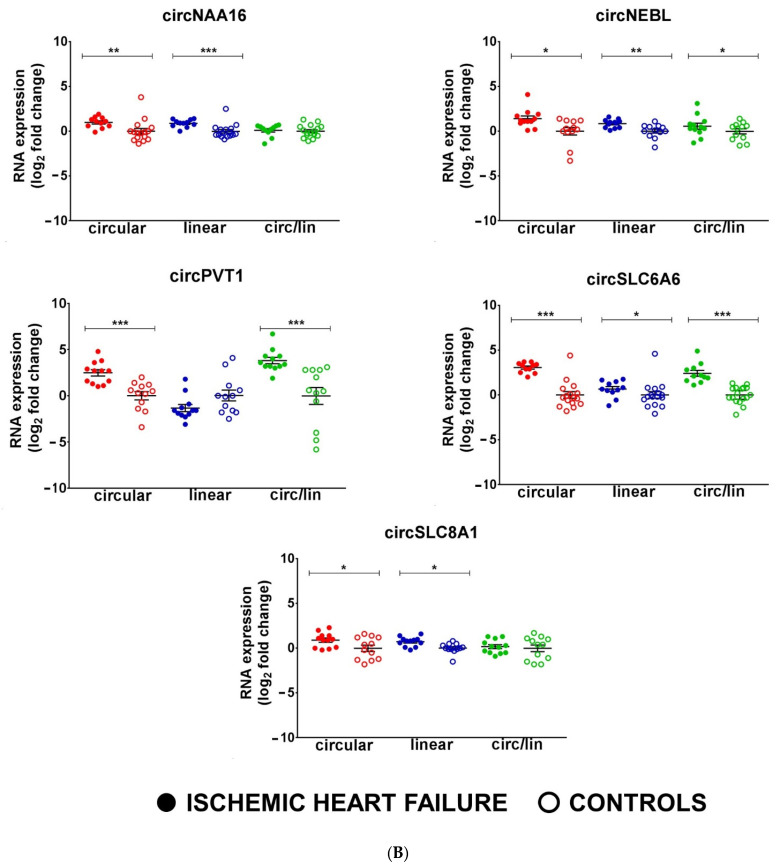

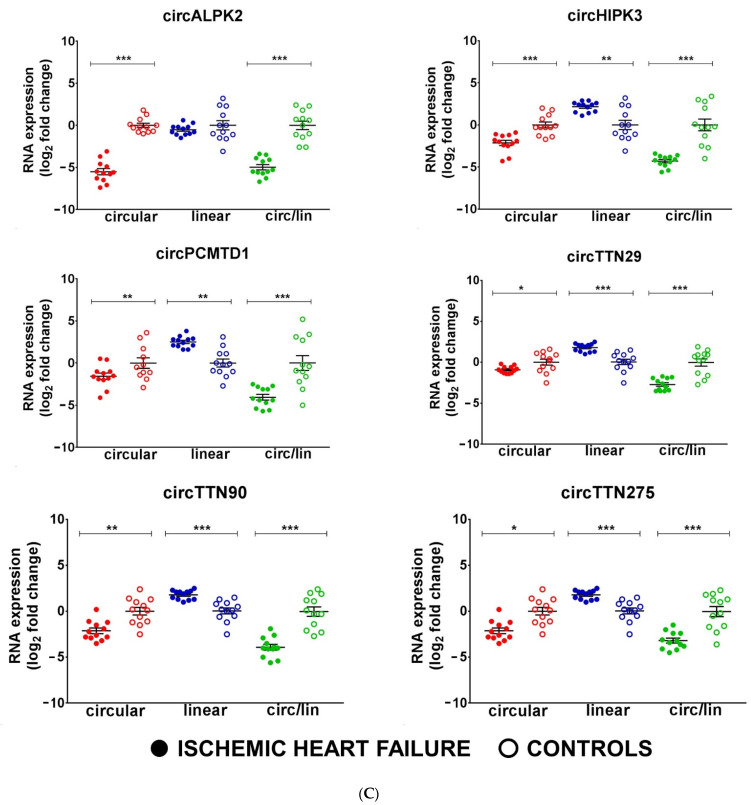
circRNAs are deregulated in non-end-stage IHF patients. Total RNA was extracted from LV tissue from IHF patients (*n* = 12) and matched control subjects (*n* = 12). Dot-plots show circRNAs and host gene relative expression measured by qPCR and expressed as log_2_ fold change. The circ-to-linear ratio (circ/lin) indicates the circRNA enrichment compared to the host gene (linRNA). (**A**,**B**) show upregulated circRNAs, while (**C**) shows downregulated circRNAs. Mean values and error bars are indicated (* *p* < 0.05, ** *p* < 0.01, and *** *p* < 0.001).

The same RNA-Seq dataset used for mRNA analysis was also employed to investigate circRNA expression, and bioinformatics analysis identified 134,713 unique back-splice sites across all libraries. A stringent abundance filter of 5 reads in at least 50% of the samples was applied, obtaining 1,500 back-splice junctions (Supplementary Table S5A) and, using |1| log_2_ fold change as expression and *p* < 0.05 as statistical significance thresholds, 213 DE back-splice junctions were identified (Supplementary Table S5B). RT-qPCR assays using divergent primers (Supplementary Table S2) validated the upregulation of circHDAC9, circMLIP, circNAA16, and circSLC6A6 in IHF patients compared to controls (Figure 3A, B). However, the DE of other circRNAs, such as circEMILIN2, circFOXP1, and cTTN, was not validated. Among the IHF DE circRNAs, prioritizing specificity over sensitivity, only qPCR-validated circRNAs were considered for further analysis.

### 3.5. Expression of Candidate circRNAs in Non-End-Stage IHF Patients

In order to complement the unbiased strategy of RNA-seq, 16 circRNAs well expressed in the heart (circALPK2, circATP2B4, circFBXO7, circHIPK3, circLAMA2, circMYBPC, circNEBL, circPCMTD1, circPRDM5, circRYR2, circSLC8A1, circTTN1, circTTN29, circTTN90, circTTN275, circTTN375) were identified from previously published data. Those circRNAs are modulated in small groups of ischemic and non-ischemic end-stage dilated cardiomyopathies [33,36]. In addition, three circRNAs already characterized in other cell systems and conditions were identified as relevant for IHF, in particular circBPTF, circPVT1, and circANKRD17 [65]. The expression levels of the circular and linear transcripts of the candidate circRNAs were measured by qPCR in 12 LV samples of non-end-stage IHF patients and 12 healthy controls (Supplementary Table S1C). All of the 19 circRNA candidates were found to be readily detectable in heart tissue; six of them showed a significant up-regulation in HF compared to controls (circANKRD17, circBPTF, circFBXO7, circNEBL, circPVT1, and circSLC8A1) (Figure 3A,B), and six were significantly down-regulated (circALPK2, circHIPK3, circPCMTD1, circTTN29, circTTN275, and circTTN90) (Figure 3C), while the other seven were not significantly modulated.

Interestingly, in most cases, the linear counterparts of the identified circRNAs were not altered to the same extent or even in the same direction of the circRNAs. Indeed, except for *SLC8A1* and *NAA16*, the circRNA-linear RNA ratio showed similar modulation of circRNA alone (Figure 3), suggesting that the DE of circRNAs was, in most cases, independent of the transcriptional regulation of their host gene.

The circRNAs identified with targeted (this paragraph) and unbiased (previous paragraph) strategies, together, provided an IHF circRNA signature for further use in the design of the network.

### 3.6. IHF ceRNA Regulatory Network

According to the ceRNA model [66], circRNAs and mRNAs sharing the same miRNA-binding sites can function as ceRNAs, thereby regulating the bioavailability of the common miRNA [27]. MRE analysis performed by CircInteractome, CircAtlas, and ENCORI identified potential interacting miRNAs for nine circRNAs. At the same time, no match was found for circHDAC9, circALPK2, circMLIP, and the circRNAs originating from the TTN gene that were either not annotated in circBase [67] or not represented in the RISC-immunoprecipitation dataset considered by ENCORI. Then, assuming that a circRNA-miRNA interaction may lead to a negative regulation of the miRNA [29,30,31], the identified miRNAs were filtered for the IHF DE miRNAs (Supplementary Table S3B), displaying an inverse modulation with the targeting circRNA and resulting in 15 DE IHF circRNA-interacting miRNAs. Interestingly, only 5 IHF DE miRNAs displayed a concordant modulation with the potentially corresponding circRNA, corroborating the assumption of a predominant negative miRNA-circRNA interaction.

**Figure 4:**
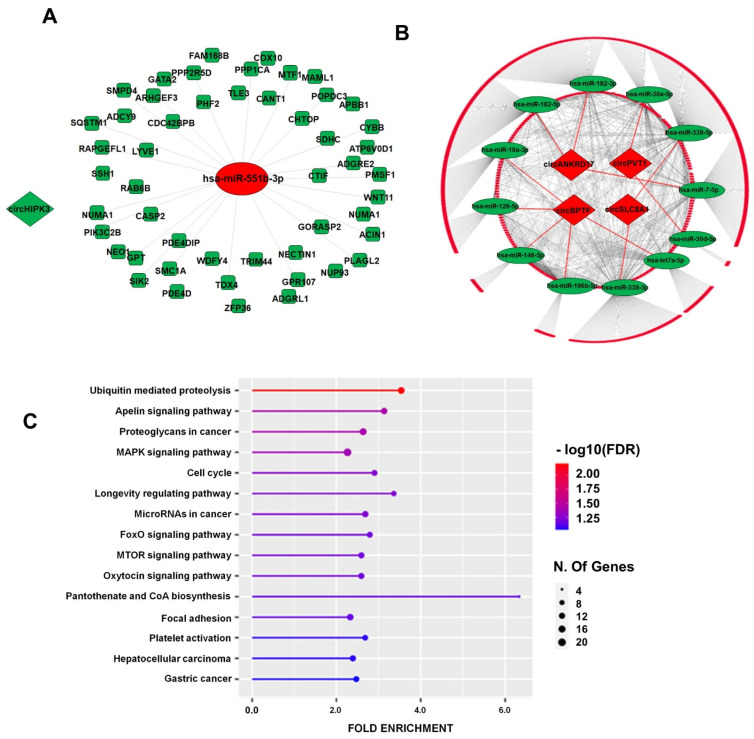
circRNA-driven circRNA–miRNA–mRNA regulatory network in IHF. circRNAs are indicated as diamonds, miRNAs as ovals, and mRNAs as rounded rectangles; green or red colors indicate down- or up-regulation in IHF, respectively. (**A**) The network is driven by down-regulated circHIPK3. (**B**) The network is driven by the up-regulated circRNAs; the inner circle is constituted by mRNAs targeted by multiple miRNAs, and the outer circle displays single miRNA-mRNA interactions (for details on mRNAs in the network, please refer to Supplementary Table S6). (**C**) The top 15 KEGG terms of the identified interacting mRNAs are represented by a lollipop graph and ordered by FDR enrichment. The size of the lollipop indicates the number of genes, and colors indicate the statistical significance (red for lower FDR, blue for higher FDR).

The mRNA target analysis of the circRNA-interacting miRNAs by MirDIP (https://ophid.utoronto.ca/mirDIP/) identified 2,566 mRNAs, and the overlap of this list with the dataset of the mRNAs modulated in IHF (Figure 2) identified 13 miRNAs and 662 inversely modulated mRNAs targeted by these miRNAs to be considered for further analysis (Supplementary Table S6 and Table 1). Finally, the resulting 662 HF-circRNA-miRNA-mRNA interactions were used for the ceRNA network generation (Table 1, Supplementary Table S6). The network was visualized by Cytoscape 3.8.2 and included 5 circRNAs, 13 miRNAs, and 662 mRNAs (Table 1; Figure 4 A,B).

The functional enrichment analyses of KEGG terms and the Gene Ontology (GO) of the modulated targets were performed to gain insights into the functional relevance of the identified network. Figure 4C and Supplementary Table S7A,B show that, among the top statistically significant enriched KEGG terms, there were several pathways related to HF disease mechanisms, such as Ubiquitin-mediated proteolysis, Apelin signaling, and Mammalian Target of Rapamycin (MTOR) signaling pathways. Supplementary Figure S1 provides KEGG pathway diagrams of some of these enriched pathways, where enriched mRNAs are highlighted in red. Accordingly, both the GO Biological Process and the GO Molecular Function analyses indicated the “Ubiquitin-related terms” among the top 10 enriched terms (Supplementary Table S7C,D), confirming the KEGG analysis.

Summarizing these findings, a network including IHF DE circRNAs, miRNAs, and mRNAs was generated, and GO and KEGG terms relevant for IHF pathogenesis were identified.

### 3.7. Molecular Characterization of circBPTF

Among the circRNAs of the HF ceRNA regulatory network, circBPTF was selected for further characterization according to (i) the readily detectable expression in LV samples, (ii) the extent of the related increase in failing hearts compared to controls, and (3) the circular/linear ratio fold increase in IHF. Moreover, circBPTF displayed the highest number of interactions with miRNAs in the identified ceRNA network.

CircBPTF is a circRNA that is formed by reverse splicing from exons 21 to 27 of the gene encoding *bromodomain PHD finger transcription factor* (*BPTF*) located on chromosome 17, and a circRNA with this junction is annotated in circBase [67] as hsa_circ_0000799 (Figure S2A). In order to further characterize the structure of circBPTF, the back-splicing junction was verified by Sanger sequencing in LV-derived RNAs (Figure S2A). Briefly, seven divergent primer couples were used to amplify each of the predicted circBPTF exons. The fragment size and sequence were analyzed by agarose gel and Sanger sequencing. The circBPTF isoform expressed in the heart had a structure similar to hsa_circ_0000799, differing only for a shorter form of exon 21 (Figure S2A). Specifically, a 493-base form of exon 21 was included (ENSE00002506735), as opposed to the 917-base form annotated in circBase (ENSE00001223457) (Figure S2A,B). The same exon composition and back-splicing junction were identified in the myocardium of both IHF and controls. 

The resistance to the RNase R exonuclease of the identified circBPTF transcript was assayed in order to test whether the transcript was circular [68]. The LV RNA amplified using the divergent primers was almost wholly resistant to the exonuclease, while the host mRNA was partially accessible (Figure S2C). Moreover, nuclear/cytoplasmic cellular fractionation in the AC16 cardiomyocyte cell line indicated that circBPTF was preferentially located in the cytoplasm, while the linear form was distributed to similar levels in the cytoplasmic and nuclear fractions (Figure S2D).

### 3.8. Increased circBPTF Levels in End-Stage IHF

**Figure 5:**
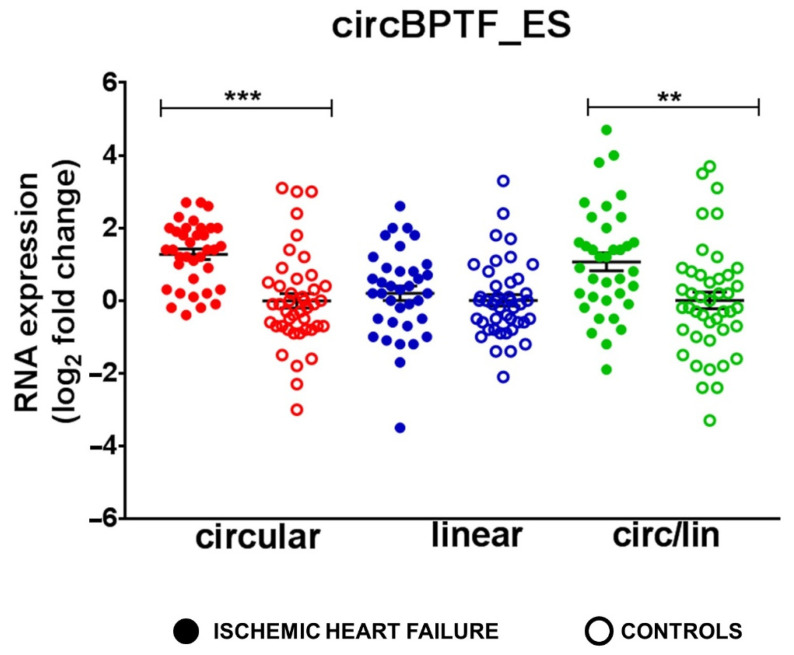
Increased circBPTF levels in end-stage IHF patients. Total RNA was extracted from LV tissue derived from end-stage IHF patients (*n* = 36) and matched control subjects (*n* = 44). Dot-plots show circRNAs and host gene relative expression measured by qPCR and expressed as log_2_ fold change. Circ-to-linear ratio (circ/lin) indicates the circRNA enrichment compared to the host gene (linRNA). Mean values and error bars are indicated (** *p* < 0.01 and *** *p* < 0.001).

As a means to further validate circBPTF modulation in IHF, we tested the respective levels in patients affected by end-stage IHF undergoing heart transplantation. RNA was extracted from the remote LV area of 36 end-stage IHF patients and 44 healthy controls (Supplementary Table S1C). Figure 5 shows that, while circBPTF levels were increased in end-stage IHF, the levels of linear BPTF were not altered significantly. Accordingly, the circRNA-linear RNA ratio was also increased in IHF. These results are in accordance with the modulation of circBPTF in non-end-stage IHF patients.

### 3.9. circBPTF Levels Are Induced by Hypoxia in Endothelial Cells

**Figure 6:**
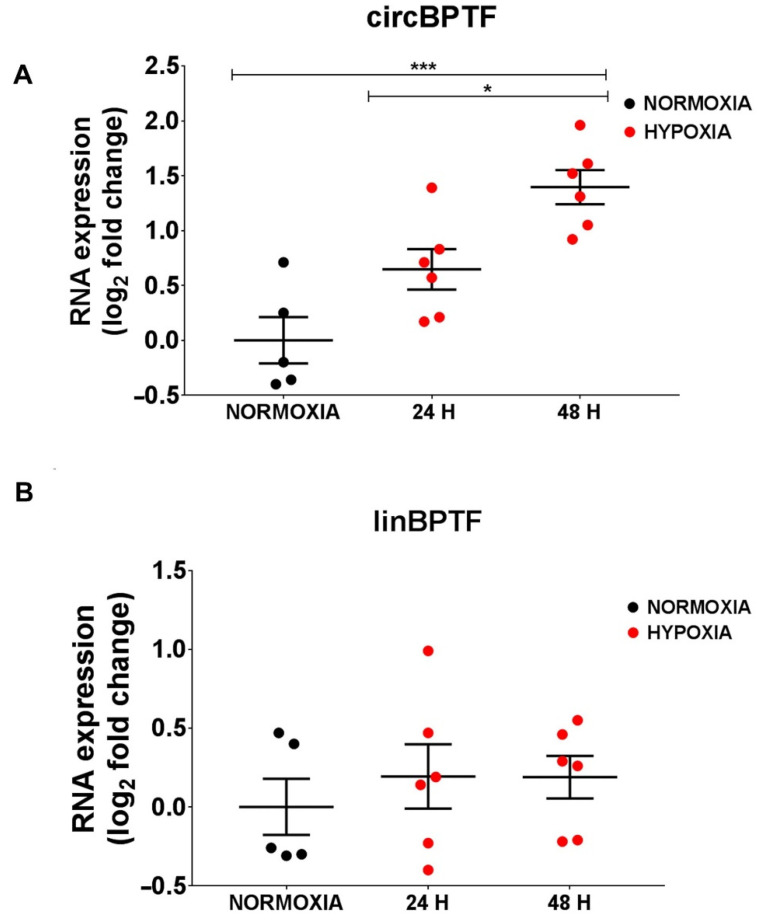
circBPTF induction by hypoxia in endothelial cells. HUVEC were cultured in 1% hypoxia or normoxic conditions for 24 or 48 h, and the expression levels of circBPTF (A) and linBPTF (B) were measured by RT-qPCR and expressed as log_2_ fold change. Only the expression of the BPTF circular form (A) was significantly increased in hypoxic conditions. Mean values and error bars are indicated (* *p* < 0.05 and *** *p* < 0.001).

Hypoxia and inflammation are pathogenetic stimuli in IHF [69,70,71,72]. First of all, we tested whether circBPTF levels were modulated by hypoxia in relevant myocardial cell types such as endothelial cells, cardiomyocytes, and cardiac fibroblasts. The expression levels of the circular and linear forms of *BPTF* were measured in HUVEC endothelial cells, the AC16 myocardial cell line, and HCF cardiac fibroblasts exposed to 1% hypoxia or normoxic conditions. In endothelial cells, hypoxic stress induced circBPTF expression already at 24 h and further increased at 48 h of culture (Figure 6A). The expression level of the linear counterpart did not change at both time points (Figure 6B), indicating a modulation specific to the circular form. Conversely, in AC16 cells and cardiac fibroblasts, circBPTF levels were only marginally or not modulated by low oxygen (Figure S3).

Next, the effects of inflammatory stimuli were tested in HUVEC using two doses (2.5 and 10 ng/mL) and two incubation times (8 and 24 h) of interleukin-1 β (IL1β) and of Lipopolysaccharide (LPS), the most abundant component within the cell wall of Gram-negative bacteria. IL1β has a well-established pro-inflammatory role in cardiovascular diseases [73,74]. The stimulation with LPS, which is not involved in IHF, represents a good model for studying *in vitro* cellular inflammatory responses.

Total RNA from treated and not-treated (CONTROL) cells was extracted, and circBPTF, linBPTF, as well as *COX2* (*PTGS2*) [75,76], *ICAM1*, *VCAM1* [77,78], *IL6* [79], and *IL8* [80], markers of the inflammatory response, were measured. As expected, LPS and IL1β treatments, at both doses and incubation times, increased *COX2, ICAM1, VCAM1, IL6*, and *IL8* (Figure S4A–D). Conversely, neither LPS nor IL1β increased circBPTF levels at any tested dose or incubation time (Figure S4E–H). In particular, reduced expression of both circular and linear *BPTF* was observed upon IL1β incubation, indicating a modulation that was not specific to the circular isoform.

These findings show that the circBPTF pathway was responsive to hypoxia mainly in endothelial cells, indicating this cell type as a relevant experimental system [69,70].

### 3.10. Validation of circBPTF as a Functional Element of the IHF circRNA-miRNA-mRNA-ceRNA-Network

**Figure 7:**
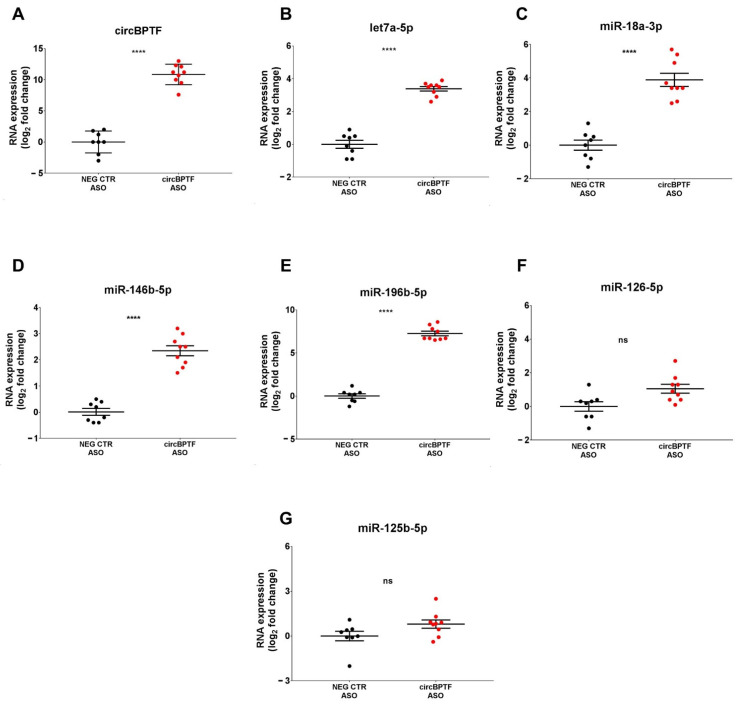
Let-7a-5p, miR-18a-3p, miR-146b-5p, and miR196b-5p enrichment in circBPTF pull-down. Biotinylated ASOs targeting the circBPTF back-splicing junction (circBPTF ASO) or biotinylated non-targeting control oligonucleotides (NEG CTR ASO) were used in pull-down experiments of HUVEC lysates. RNA bound to streptavidin-coated beads was extracted, and the levels of (**A**) circBPTF or (**B**–**F**) the miRNAs identified as interactors of circBPTF or (**G**) miR-125b-5p, which was not a circBPTF interactor (negative control), were assayed by RT-qPCR and expressed as log_2_ fold change. Mean values and error bars are indicated (*n* = 8, **** *p* < 0.0001). ns = not statistically significant.

A circRNA pull-down assay was used to capture the circBPTF-associated miRNAs under physiological conditions in HUVEC to validate the predicted miRNA-circRNA interactions. First of all, we checked that there were readily detectable levels in HUVEC (not shown). Then, the 5 circBPTF miRNA interactors (Table 1) were measured in a pull-down extract using an ASO targeting the circBPTF back-splicing junction (circBPTF-ASO) or a control non-targeting oligonucleotide (NEG-CTR-ASO). As expected, circBPTF was enriched in the circBPTF-ASO pull-down, confirming the efficiency of circBPTF targeting (Figure 7A). Four of the five identified miRNAs were enriched in circBPTF-ASO RNA pull-downs (Figure 7B–E), while miR-126-5p enrichment did not reach statistical significance (Figure 7F). Additionally, miR-125-5p, unpredicted to interact with circBPTF and selected as a negative control, was not enriched in circBPTF-ASO pulldown (Figure 7G).

The CircBPTF/miR-196b-5p interaction was explored for further validation of the ceRNA network. First of all, we tested whether, in a reverse approach, circBPTF was enriched in miR-196b-5p pull-down extract. Figure S5 shows that biotinylated miR-196b-5p was enriched in miR-196b-5p biotin pull-down, confirming the technique’s efficiency (Figure S5A). CircBPTF was highly enriched in biotinylated miR-196b-5p pull-downs, while non-targeted circRNAs circARHGAP10 and circARGHEF12 were not significantly enriched (Figure S5B).

Thereafter, we assessed whether circBPTF silencing interfered with the miRNA-mRNA targeting process, decreasing miR-196b-5p targets. To this end, two small interfering RNAs (siRNAs) targeting the head-to-tail junction of circBPTF were designed (Supplementary Table S2B). The relative expression of the circular and linear forms was measured, and both siRNAs, with comparable efficiency, specifically downregulated the circular form without affecting the host mRNA (Figure S6A). Then, we assayed two miR-196b-5p targets, *HDAC9* and *LRRC17*, selected for their easily detectable levels in endothelial cells [81]. As predicted in a “sponge effect” setting, circBPTF silenced by siRNAs#1 and #2 reduced the expression of *HDAC9* and *LRRC17* (Figure S6B,C).

**Figure 8:**
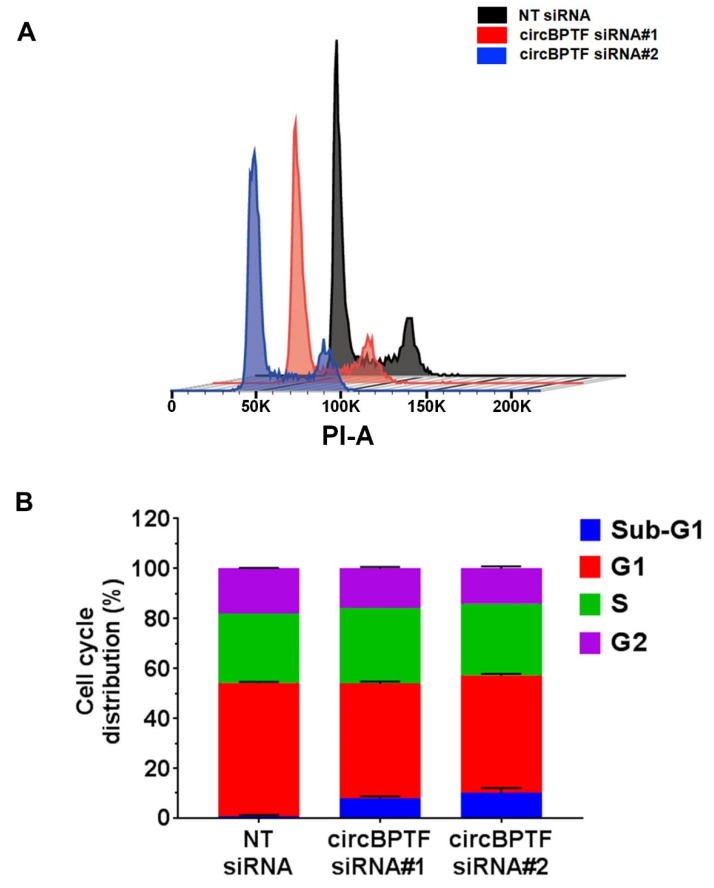
HUVEC proliferation inhibition by circBPTF silencing. Two siRNAs targeting the back-splice junction of circBPTF and a non-targeting siRNA control (NT siRNA) were transfected in HUVEC, and after three days, cell numbers were counted or flow cytometry cell analysis was performed. (**A**) Representative cell cycle profiles obtained by flow cytometry. (**B**) Quantitative analysis of the proportion of cells in each phase. A statistically significant decrease of the G1 (*p* < 0.001) and G2 (*p* < 0.01) phases and an increase of sub-G1 cells (*p* < 0.001) were observed in cells treated with both circBPTF siRNAs compared to non-targeting siRNAs. The bar graph shows the mean values and standard error of the data (*n* = 3).

circBPTF, by interacting with the identified miRNAs, could be a potential indirect regulator of the expression of 220 mRNA targets (Table 1 and Supplementary Table S6). A functional enrichment analysis of KEGG and REACTOME terms of these targets indicated several pathways related to HF disease mechanisms, including those regulating cell cycle and apoptosis (Supplementary Table S8). Thus, we tested whether circBPTF silencing affected endothelial cell proliferation. Figure S7 shows that cell number was significantly decreased in cells transfected with circBPTF siRNAs#1 or #2 compared to non-targeting siRNA control, starting at 48 h for both conditions (Figure S7A,B). Moreover, flow cytometry cell cycle analysis showed a statistically significant decrease in the percentage of cells in G1 and G2 phases and a higher proportion of sub-G1 cells, which was indicative of increased cell death, in cells treated with both circBPTF siRNAs compared to non-targeting siRNAs (Figure 8A,B).

These data confirm the identification of a functionally active circBPTF-miRNAs-mRNAs network in IHF.

## 4. Discussion

Data on circRNA-miRNA-mRNA networks in IHF are still limited, primarily restricted to end-stage IHF patients [40,41,42]. In this study, the aim was to establish a large-scale regulatory network based on the ceRNA theory involving multiple types of RNAs, including circRNAs, miRNAs, and mRNAs, identified in non-end-stage IHFs patients. To this purpose, we have employed a multipronged approach, combining bioinformatics analysis of RNA sequencing datasets with qPCR validation.

CircRNA profiling is a potent approach for defining the circRNA expression pattern. However, some of the limitations of this approach are the need for very deep sequencing data, the incomplete genome annotation, and the lack of consensus in bioinformatics tools for data analysis [17]. Deregulated circRNAs validated with an orthogonal technique (qPCR) were selected in order to increase specificity in the analysis of IHF LV samples. This strategy narrows the complexity of the network compared to others, but it makes the network more likely to be biologically relevant.

To complement the unbiased strategy of RNA-seq, the deregulation in non-end-stage IHF patients of circRNAs found to be modulated by previously published studies in small groups of both ischemic and non-ischemic end-stage dilated cardiomyopathies [33,36] or characterized in other cell systems and conditions that are relevant for IHF, such as senescent fibroblasts [82], was evaluated. Validated circRNAs were considered for further analysis of IHF in order to increase specificity. Indeed, the comparison of circRNA changes among studies is hampered by several aspects. For example, the lack of a unique nomenclature, the limited number of enrolled patients, and/or the different dilated cardiomyopathy etiologies (e.g., ischemic vs. non-ischemic or end-stage vs. non-end-stage IHF). Interestingly, in line with the work of Tan et al., based on RNA-Seq data from three IHF patients and three controls only, we observed increased levels of circFBXO7, circNEBL, and circSLC8A1 and decreased levels of circPCMTD1 in IHF LV [33]. The up-regulation of circSLC8A1 and circBPTF together with the down-modulation of circALPK2 previously observed in a limited number of patients and different etiologies [34,35,36,83] were also confirmed.

Very interestingly, we also observed the down-modulation of circTTN29, circTTN90, and circTTN275, which are generated from a specific region within the I-band of the *TITIN* gene encoding for a large protein responsible for the passive elasticity of the muscle. This specific region is also affected by extensive alternative splicing that produces several *TITIN* isoforms [84] and generates the most significant number of circRNAs per gene in the heart [34,36]. The results are consistent with two earlier studies reporting similar dysregulation in dilated cardiomyopathies [36,37].

After an articulated pipeline employed by the bioinformatics analysis to intersect experimental data, we identified circRNA-miRNA-mRNA interactions formed by 5 circRNAs (circANKRD17, circBPTF, circPVT1, circSLC8A1, and circHIPK3), 13 miRNAs, and 662 mRNAs. It is unlikely that other interactors may give the same result unless they share the same experimentally validated targets as those identified by this analysis.

Only a part of the 16 candidate circRNAs were used for ceRNA network generation. Indeed, the use of the circRNA-miRNA interaction databases requires the circRNA to be annotated in circBase [67], CircInteractome [51], and/or CircAtlas [52], but also the binding sites to be enriched in Argonaute Clip-Seq data (ENCORI) [50]. In particular, circTTN275, circTTN29, circTTN90, and circALPK2 did not satisfy these requirements. Other circRNAs were excluded because of the stringent analysis pipeline that imposed multiple experimental and analytical filters. A fundamental selection criterion was constituted by the fact that only counter-modulated circRNA-miRNA couples were considered. In particular, in most cases, miRNA targeting by circRNA results in a reduction of miRNA levels [29,30,31], but this is not always the case [85,86]; the additional filtering criterion maximized specificity at the expense of sensitivity.

mRNA targets from the identified ceRNA network were further analyzed for the functional enrichment analysis of KEGG terms, and Apelin, mTOR, and ubiquitin pathways resulted among the top-15 enriched KEGG terms (Figure 4C and Figure S1). Specifically, Apelin is a ligand of the “orphan” G-protein-coupled receptor Angiotensin-like 1 receptor (APJ), and this interaction inhibits cardiac fibrosis by suppressing the TGF-beta pathway [87,88]. Indeed, Transforming Growth Factor Beta Receptor 1 (TGFBR1) and SMAD2/4 are enriched in that pathway, as mRNAs target the interactions circSLC8A1/miR-338, circANKRD17/miR-182-3p, and circBPTF/let-7a-5p. Both TGF-beta and SMAD2/4 have a well-recognized role in heart fibrosis [89,90,91]; likewise, miR-338-3p and let-7a regulate cardiac fibrosis and hypertrophy [92,93,94,95], while miR-182 has a role in pulmonary and liver fibrosis [96,97].

Other ceRNA interactions with relevance in cardiovascular pathology are circANKRD17/miR-182, circPVT1/miR-30a/d-5p, and circSLC8A1/miR-338-3d interactions with the *Frizzled* (*FZD*) mRNAs, enriched in the mTOR signaling pathway (Figure S1). The Wnt-Frizzled (Fzd) proteins are G-protein-coupled receptors involved in Wnt signaling. Increasing evidence reports that Wnt-Fzd signaling is important in cardiac hypertrophy, fibrosis, myocardial infarction, and arrhythmias [98,99].

Autophagy and the ubiquitin-proteasome system (UPS) are the most critical degradation mechanisms controlling protein homeostasis systems. Impairment in cardiac proteasomal degradation and autophagy are associated with several cardiovascular diseases, including HF [100,101,102]. Accordingly, we have found 16 interactions of ubiquitin system members with all of the upregulated circRNAs in the ceRNA network.

In order to experimentally validate the circRNA-miRNA interactions and their consequences on the miRNA targets in the network, we have selected circBPTF among the top up-regulated in IHF and most expressed in LV. First of all, the deregulation of circBPTF in IHF was confirmed by measuring the respective levels in an independent group of IHF patients undergoing heart transplantation, showing that the circBPTF increase is not limited to non-end-stage HF. After that, endothelial cells, the fundamental cell component of the heart, were identified to display increased circBPTF levels when exposed to a relevant stress, such as hypoxia. Conversely, inflammatory stimulation by LPS or IL1β did not induce circBPTF levels, indicating hypoxia as a main stress effector.

Different network levels were tested experimentally. Pull-down assays confirmed the validity of most miRNA-circBPTF interactions. Interestingly, among the enriched miRNAs, let-7a-5p and miR-18a-3p are implicated in pathological mechanisms leading to HF [94,95,103,104]. Moreover, silencing circBPTF resulted in the expected deregulation of miRNA target gene expression. Pathway enrichment analysis of the mRNA targets controlled by the circBPTF-interacting miRNAs indicated several relevant enriched terms. Among these, we experimentally showed that the perturbation of circBPTF levels in endothelial cells triggered cell cycle arrest in the G1/G2 phase and decreased survival [67,92,93]. These observations agree with previous ones, indicating a pro-proliferative function of circBPTF in different experimental settings [105,106]. However, in HUVEC cultured in high glucose, circBPTF silencing rescued cell apoptosis, protecting against high glucose-induced inflammatory injuries and oxidative stress, indicating a context-dependent function [107].

In conclusion, this study identified dysregulated circRNAs, miRNAs, and mRNAs in IHF and explored their potential relationships and functions. A circRNA-driven circRNA-miRNA-mRNA network was created following the ceRNA theory, with circBPTF emerging as one of the main hubs. The experimental validation of these interactions supports the biological functionality and accuracy of the proposed network.

However, this study presents some limitations. The focus was exclusively on RNA species, even though circRNAs can also interact with RNA-binding proteins [32]. Additionally, the rigorous experimental approach, which includes stringent selection criteria, may prioritize specificity at the expense of sensitivity. Insufficient annotation of the circRNAs and of their interactions, as well as the use of orthogonal techniques in the analysis, both contributed to limiting the sensitivity. Indeed, the network was generated to identify the most probable RNA interactions in our model and validate the most interesting ones, without aiming at constructing a global model with high robustness and sensitivity to all possible parameters in network construction. Nevertheless, this study unveiled valuable RNA-based molecular insights into IHF pathogenesis, offering a new perspective on the disease and setting the stage for further research into circRNAs role in developing IHF.

## Figures and Tables

**Table 1:** circRNA-miRNA-mRNA interactions.

circRNA	miRNA	662 circRNA-miRNA-mRNA Interactions
circANKRD17	hsa-miR-7-5p	124
	hsa-miR-182-3p	
	hsa-miR-182-5p	
circBPTF	hsa-let-7a-5p	220
	hsa-miR-18a-3p	
	hsa-miR-126-5p	
	hsa-miR-146b-5p	
	hsa-miR-196b-5p	
circHIPK3	hsa-miR-551b-3p	50
circPVT1	hsa-miR-30a-5p	155
	hsa-miR-30d-5p	
circSLC8A1	hsa-miR-338-3p	113
	hsa-miR-338-5p	

## Data Availability

RNA-sequencing raw and processed data were uploaded to GEO: GSE198945 (https://www.ncbi.nlm.nih.gov/geo/query/acc.cgi?acc=GSE198945, accessed on 14 November 2022; ask the authors for consultation before data release).

## Supplementary Materials

The following supporting information can be downloaded at: https://www.mdpi.com/article/10.3390/cells12212578/s1, Figure S1: APELIN, mTOR and Ubiquitin mediated proteolysis KEGG diagrams; Figure S2: Characterization of circBPTF in the heart.; Figure S3: circBPTF in hypoxic AC16 cells and heart fibroblasts; Figure S4: Effects of IL1β and LPS treatment on circBPTF expression in HUVEC; Figure S5: Specific circBPTF enrichment in miR-196b-5p pull-down; Figure S6: circBPTF silencing reduced the expression of two miR-196b-5p mRNA targets in HUVEC cells; Figure S7: HUVEC proliferation inhibition by circBPTF silencing. Table S1: IHF patients and healthy controls characteristics; Table S2: Primers, oligonucleotides and siRNA sequences used in the experiments; Table S3: IHF differentially expressed miRNAs; Table S4: IHF differential expressed mRNAs and Gene Ontologies enriched terms; Table S5: IHF differentially expressed circRNAs; Table S6: IHF circRNA-IHF miRNA-IHF mRNA interactions; Table S7: KEGG pathways and Gene Ontology enriched terms of ceRNA network; Table S8: KEGG and Reactome pathways enriched terms in circBPTF network. References [67,108,109] are cited in the supplementary materials
