# Supplementary material for: circRNA-miRNA-mRNA Deregulated Network in Ischemic Heart Failure Patients
Source: Cells. 2023 Nov 5;12(21):2578. doi: 10.3390/cells12212578 (PMC10648415; doi:10.3390/cells12212578)
Supplement: Supplementary file 1 [file cells-12-02578-s001.zip › Supplemental Materials and Methods.pdf]

Supplementary Materials and Methods

RNA isolation and qPCR

Total RNA was extracted using TRIzol (Thermo Fisher Scientific Inc, Waltham, MA, USA) following manufacturer's instruction. Integrity, quantitation and quality of the extracted RNAs were assessed by Nanodrop ND-1000 (Nanodrop, Thermo Fisher Scientific Inc, Waltham, MA, USA) and Bioanalyzer 2100 (Agilent Technologies, Santa Clara, CA, USA), respectively.

For circRNAs or mRNAs qPCR, total RNA was retro-transcribed using the GoScript Reverse Transcription System (Promega, Madison, WI, USA) in the presence of random hexamers and cDNAs were analyzed using the GoTaq(R) qPCR Master Mix (Promega, Madison, WI, USA) according to the manufacturer's instructions on a StepOne Plus Instrument (Thermo Fisher Scientific Inc, Waltham, MA, USA). Divergent primers were designed according to the back-splicing junction annotated in CircBase database [67], and convergent primers were designed for the related host linear transcript. All the primers were designed using the GRCh38/hg38 genome assembly sequences (Supplementary Table S2). After normalization for the average of UBC and RPL23A expression levels, relative RNA expression was calculated using the $2^{-\Delta\Delta Ct}$ method [108]. Regarding miRNAs expression evaluation, cDNA was prepared following manufacturer's instructions using Applied Biosystems™ TaqMan™ MicroRNA Reverse Transcription Kit and miRNAs were measured using TaqMan MicroRNA single assays and TaqMan™ Universal PCR Master Mix following manufacturer's instructions (Thermo Fisher Scientific Inc, Waltham, MA, USA). All reagents were provided by Thermo Fisher Scientific Inc, (Waltham, MA, USA). Real time PCR was performed on Step-One plus real-time PCR System (Thermo Fisher Scientific Inc., Waltham, MA, USA). Sequences of designed primers are reported in Supplementary Table S2.

RNAse-R assay

For RNase R digestion assay, total RNA obtained by LV biopsies of both IHF patients and healthy controls was used. One μg of RNA was incubated with 10 U RNase R (LGC Biosearch Technologies,

London, UK) and 10 U of RiboLock RNase Inhibitor (Thermo Fisher Scientific Inc, Waltham, MA, USA) in 1× RNase R buffer in a 10 µL reaction at 37 °C for 30 min, followed by heat inactivation at 95 °C for 3 min. For comparison, the samples were also incubated under the same conditions without RNAse R.

Nuclear and cytoplasmic fractions isolation

The separation of nuclear and cytoplasmic fractions was performed in AC16 cells by using the PARIS™ Kit (Thermo Fisher Scientific Inc, Waltham, MA, USA), following the manufacturer's instructions. Next, RNA was extracted using TRIzol reagent (Thermo Fisher Scientific Inc, Waltham, MA, USA), as described above. In order to check the efficiency of the fractionation, RPL23 and MALAT1 were used as cytoplasmic and nuclear markers, respectively.

Cell cultures

AC16 human cardiomyocyte cell line (Merck KGaA, Darmstadt, Germany) was cultured in DMEM/F12 containing 2 mM L-Glutamine, 12.5% FBS and 1X Penicillin-Streptomycin Solution. HCF human cardiac fibroblast cells (Merck KGaA, Darmstadt, Germany) were maintained in PromoCell Fibroblast Growth Medium 3 (Merck KGaA, Darmstadt, Germany) containing the Supplement Mix Growth Medium Kit: Fetal Calf Serum: 0.1 ml/ml, Basic Fibroblast Growth Factor (recombinant human) 1 ng/ml and Insulin 5 µg/ml (Merck KGaA, Darmstadt, Germany) . Primary Human Umbilical Vein Endothelial pooled cells (HUVEC) (Gibco, Thermo Fisher Scientific Inc, Waltham, MA, USA) were cultured in EGM™-2 Endothelial Cell Growth Medium-2 BulletKit (Lonza, Basel, Switzerland). For hypoxia experiments, cells were incubated at 37°C in a Ruskinn hypoxia cabinet (Baker, Sanford, ME, USA) in the presence of 1% O_2 and 5% CO_2 for 24 or 48 hours and compared to cells incubated in normoxic conditions (37°C; 21% O_2; 5% CO_2). For inflammatory response experiments, HUVEC were stimulated with LPS from Salmonella typhosa (L7895-1MG, Merck KGaA, Darmstadt, Germany) or IL-1β (130-093-895, (Miltenyi Biotec, Bergisch Gladbach, Germany) at 2.5 or 10 ng/ml for 8 or 24 hours at 37°C.

Transfection experiments

circBPTF silencing was obtained transfecting cells with 50 nM short interfering RNA (siRNA) sequences designed to target the back-splice junction of the transcript (Eurofins Genomics, Ebersberg, Germany), Supplementary Table S2C). ON-TARGET plus non-targeting pool siRNA supplied by Dharmacon was used as negative control. Sub-confluent HUVEC cells were transfected using siRNA transfection reagent supplied by Santa Cruz Biotechnology (Dallas, TX, USA), according to the manufacturer's protocol. For cell number count, HUVEC cells were harvested 72 hours after transfection and trypan blue negative cells were counted by the Countess II cell counter (Thermo Fisher Scientific Inc, Waltham, MA, USA).

Cell cycle analysis

Cell were harvested 72 hours after transfection, resuspended in 0.1 ml of PBS, fixed in 2 ml of ice-cold 70% ethanol and stored at 4°C until analysis. Then, cells were centrifuged and washed two times with complete medium. Cell pellets were dissolved in 500 µl of Hanks' Balanced Salt solution (HBSS) containing 0.1 mg/ml Propidium Iodide (Merck KGaA, Darmstadt, Germany) , 0.6% NP-40 and 2 mg/ml RNAse A (Thermo Fisher Scientific Inc, Waltham, MA, USA). All samples were incubated for 30 min at room temperature in the dark. Cell cycle distribution was assessed by flow cytometry analysis of at least 10,000 cells per sample, using a BD FACSCanto™ (BD Biosciences, Becton & Dickinson, Franklin Lakes, NJ, USA) and results were analyzed by Flowjo v10.9FlowJo™ v10.9 Software (Becton, Dickinson and Company, Franklin Lakes, NJ, USA) using the Dean Jett Fox method [109].
