# Supplementary figures and images for: circRNA-miRNA-mRNA Deregulated Network in Ischemic Heart Failure Patients
Source: Cells. 2023 Nov 5;12(21):2578. doi: 10.3390/cells12212578 (PMC10648415; doi:10.3390/cells12212578)

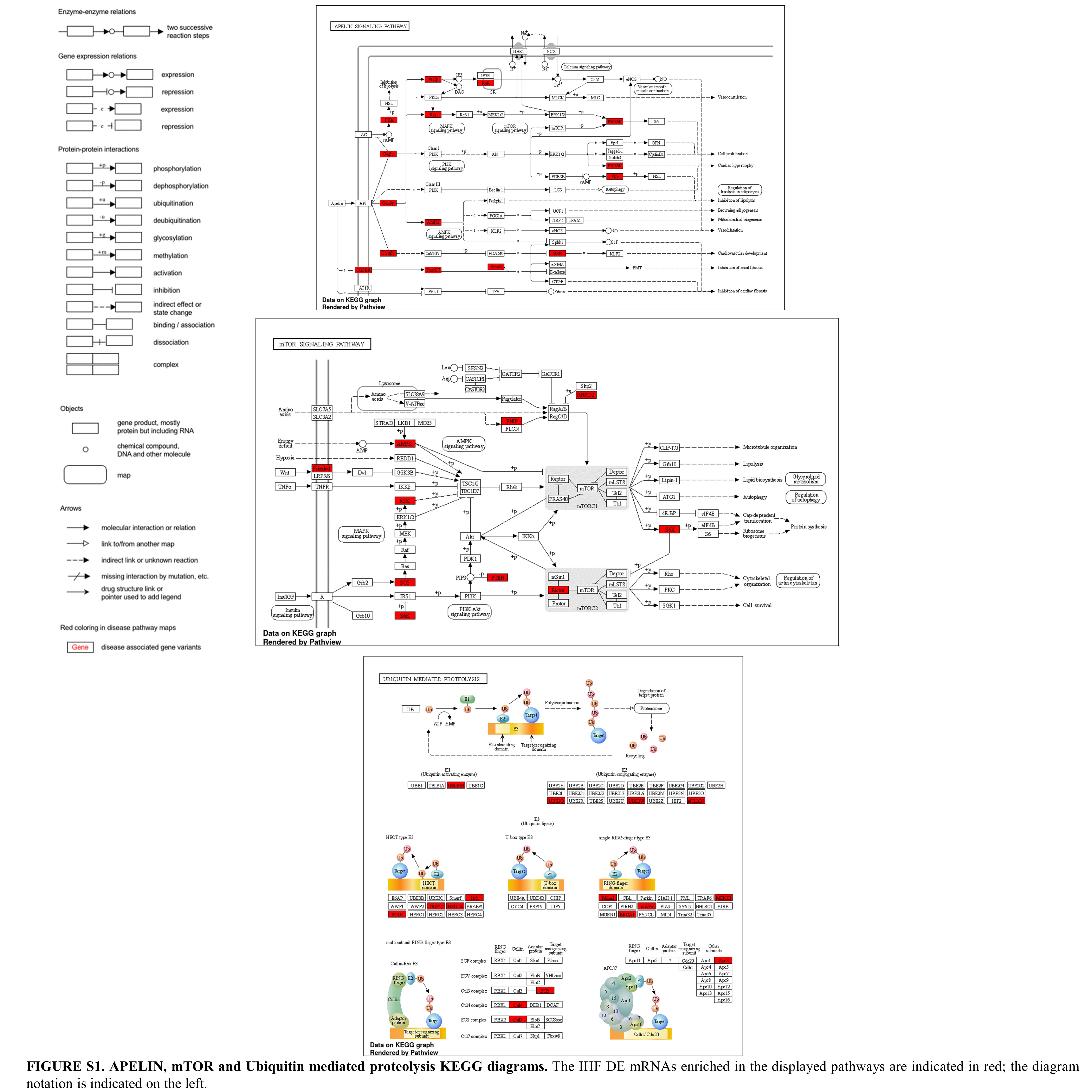

Supplement: Supplementary file 1 [file cells-12-02578-s001.zip › Supplementary Figures/Figure S1.JPG]

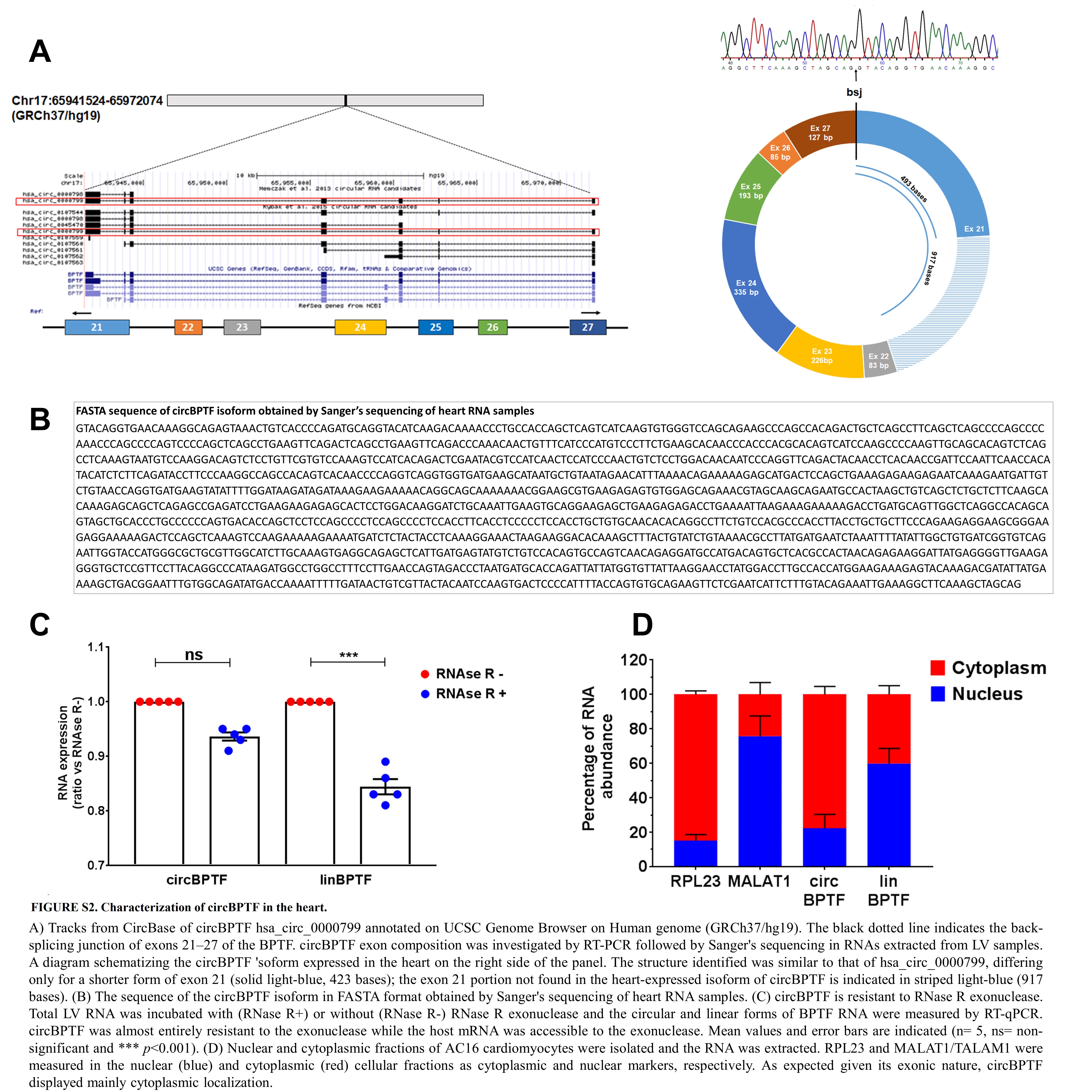

Supplement: Supplementary file 1 [file cells-12-02578-s001.zip › Supplementary Figures/Figure S2.png]

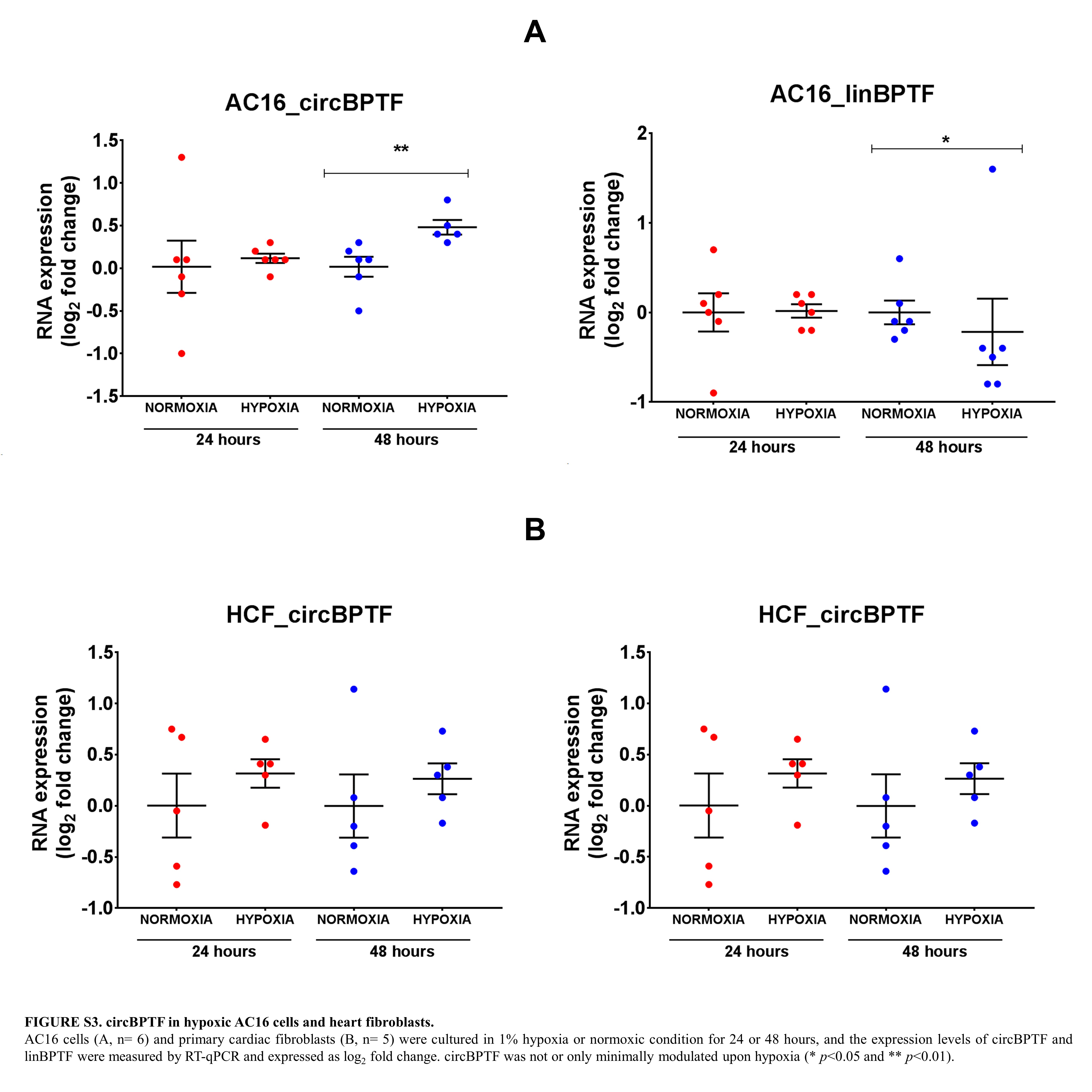

Supplement: Supplementary file 1 [file cells-12-02578-s001.zip › Supplementary Figures/Figure S3.JPG]

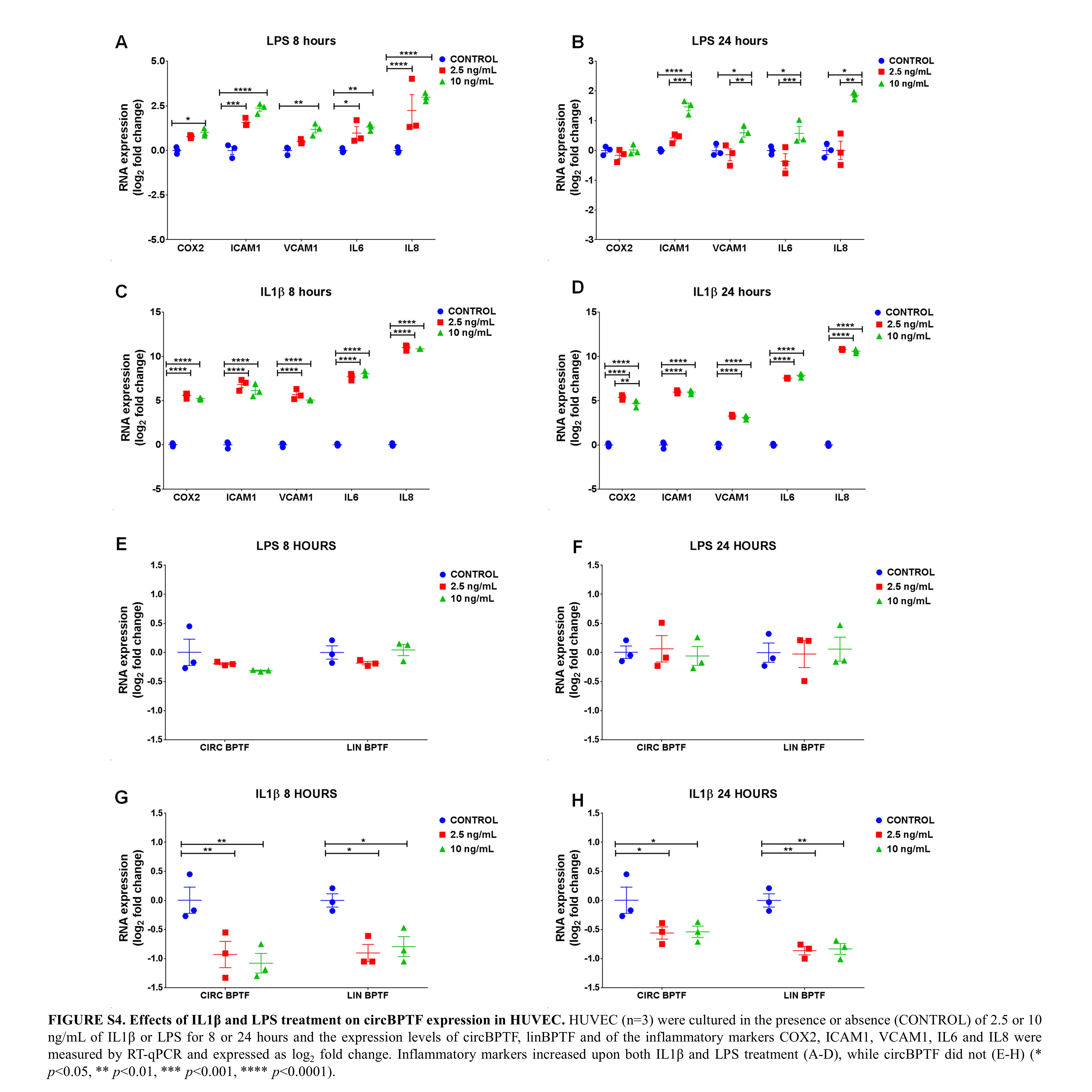

Supplement: Supplementary file 1 [file cells-12-02578-s001.zip › Supplementary Figures/Figure S4.JPG]

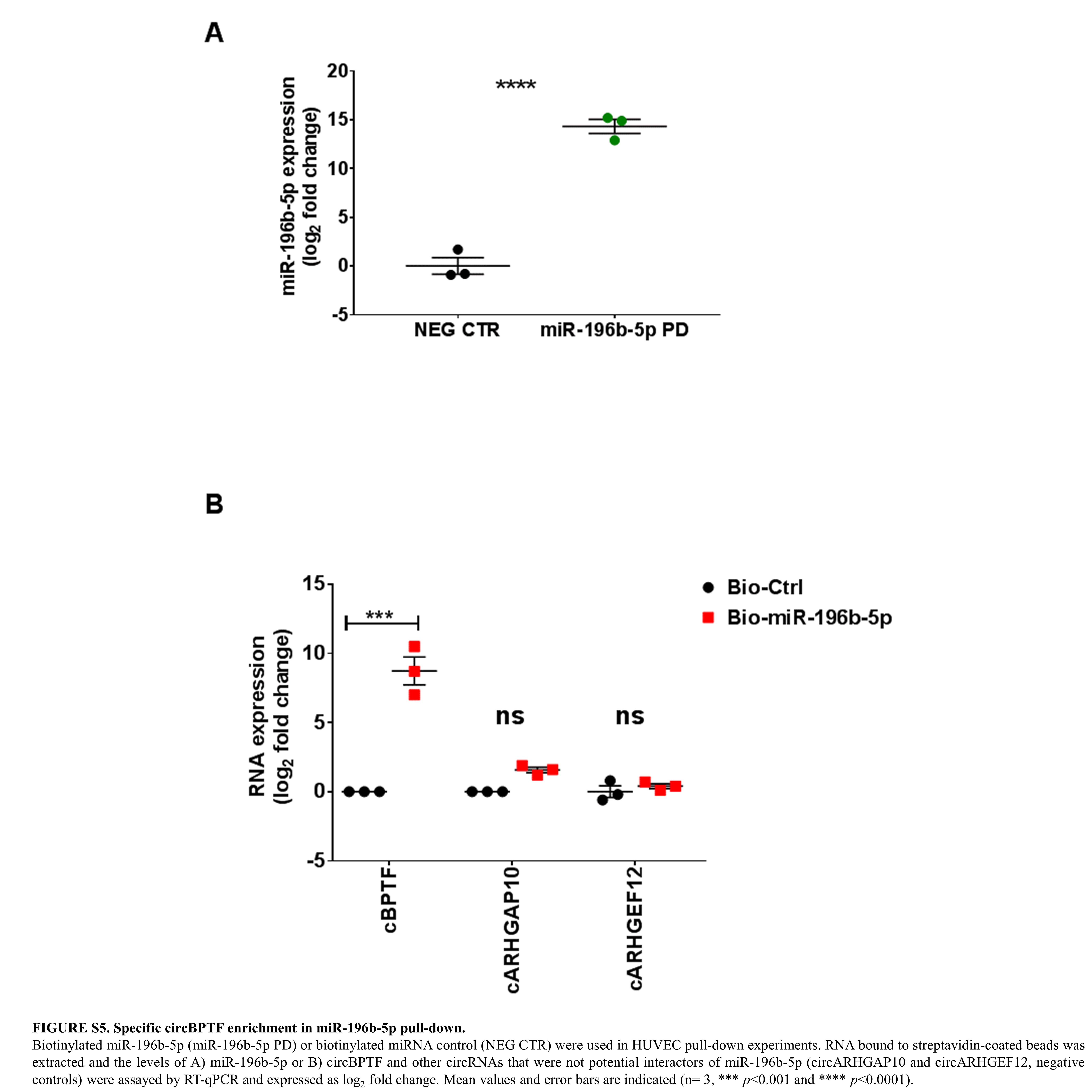

Supplement: Supplementary file 1 [file cells-12-02578-s001.zip › Supplementary Figures/Figure S5.JPG]

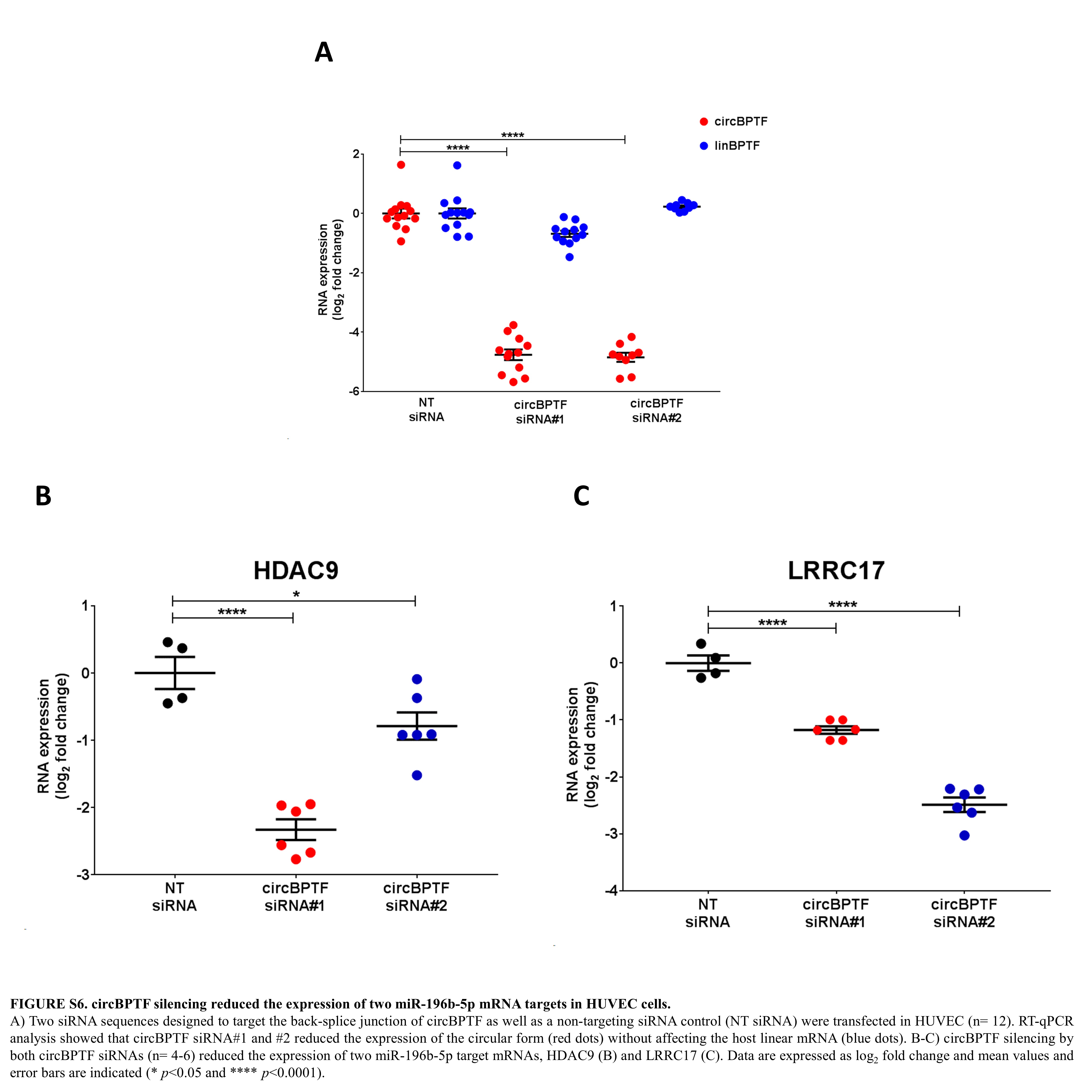

Supplement: Supplementary file 1 [file cells-12-02578-s001.zip › Supplementary Figures/Figure S6.JPG]

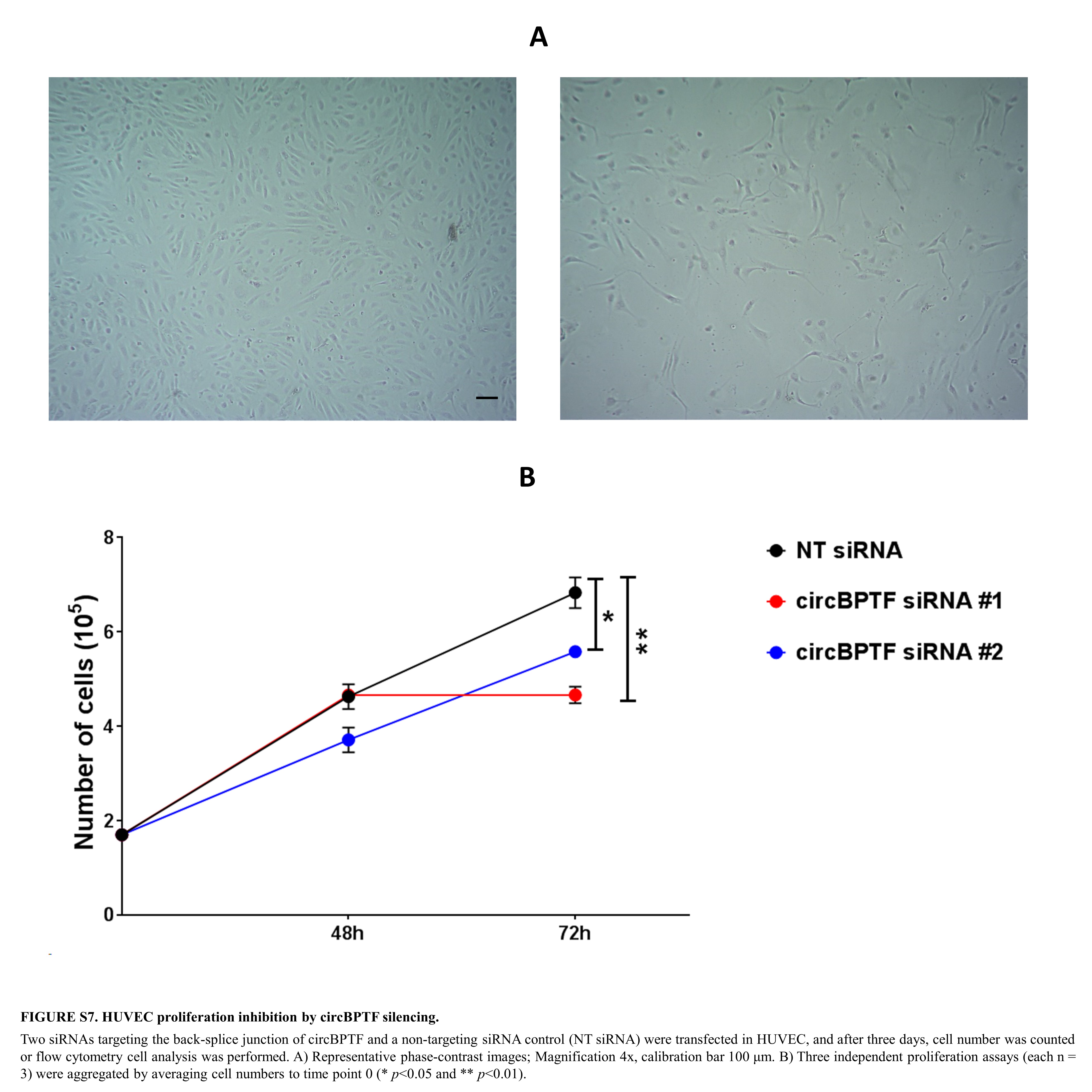

Supplement: Supplementary file 1 [file cells-12-02578-s001.zip › Supplementary Figures/Figure S7.JPG]
